# Critical appraisal in rapid systematic reviews of COVID-19 studies: implementation of the Quality Criteria Checklist (QCC)

**DOI:** 10.1186/s13643-023-02219-z

**Published:** 2023-03-27

**Authors:** Daphne Duval, Nicola Pearce-Smith, Jennifer C. Palmer, Jason Kwasi Sarfo-Annin, Paul Rudd, Rachel Clark

**Affiliations:** 1 COVID-19 Rapid Evidence Service, UK Health Security Agency (UKHSA), London, UK; 2 Research Management and Knowledge Division, UK Health Security Agency (UKHSA), London, UK; 3 Bristol Medical School, Population Health Science, University of Bristol, Bristol, UK; 4 MRC Integrative Epidemiology Unit, University of Bristol, Bristol, UK; 5 Strategic Evidence and Analysis Division, Office for Health Improvement and Disparities (OHID), Department of Health and Social Care (DHSC), London, UK

**Keywords:** Critical appraisal, Risk of bias tool, Rapid systematic reviews, Public health

## Abstract

In this letter, we briefly describe how we selected and implemented the quality criteria checklist (QCC) as a critical appraisal tool in rapid systematic reviews conducted to inform public health advice, guidance and policy during the COVID-19 pandemic. As these rapid reviews usually included a range of study designs, it was key to identify a single tool that would allow for reliable critical appraisal across most experimental and observational study designs and applicable to a range of topics. After carefully considering a number of existing tools, the QCC was selected as it had good interrater agreement between three reviewers (Fleiss kappa coefficient 0.639) and was found to be easy and fast to apply once familiar with the tool. The QCC consists of 10 questions, with sub-questions to specify how it should be applied to a specific study design. Four of these questions are considered as critical (on selection bias, group comparability, intervention/exposure assessment and outcome assessment) and the rating of a study (high, moderate or low methodological quality) depends on the responses to these four critical questions. Our results suggest that the QCC is an appropriate critical appraisal tool to assess experimental and observational studies within COVID-19 rapid reviews. This study was done at pace during the COVID-19 pandemic; further reliability analyses should be conducted, and more research is needed to validate the QCC across a range of public health topics.

**Supplementary Information:**

The online version contains supplementary material available at 10.1186/s13643-023-02219-z.

## Introduction

The COVID-19 Rapid Evidence Service at the UK Health Security Agency (UKHSA; formerly Public Health England, PHE) was set-up in April 2020 to support the use of evidence within UKHSA’s national COVID-19 response [1]. We have produced rapid reviews on a range of topics, including effectiveness of face coverings in community settings, effect of vaccination on transmission, effectiveness of interventions to reduce transmission in adult and social care settings, or risk of transmission from the deceased [2]. Our reviews focused mainly on evidence from the COVID-19 pandemic and, in the early stage, the evidence available was mostly from small descriptive epidemiological studies. As the pandemic progressed, evidence from other study designs, including cohort, case–control and interventional studies emerged. As a result, most of our rapid reviews included a range of study designs.

In the first months of the pandemic, we critically appraised studies without using formal tools based on study design and main source of bias such as selection bias, confounding, and exposure and outcome assessment. But, as the body of evidence grew and our methods evolved, we moved to implement a formal critical appraisal tool. Due to the pace at which our reviews were conducted, it was important to identify a single tool that could be used across a range of experimental and observational studies. In this letter, we describe the identification of existing critical appraisal tools, the assessment of the candidate tools, and the selection of the most relevant for use in our rapid systematic reviews. Finally, the characteristics of the tool selected are presented.

## Identification of critical appraisal tools

We conducted electronic searches on 18 January 2021 in Ovid Medline and Embase using search terms such as ‘risk of bias tool’ and ‘critical appraisal tool’, with no date limits. Results were first screened on title for relevance by an Information Scientist and 78 records reporting on critical appraisal were selected. These 78 records were screened on title and abstract by a Reviewer and records were excluded if they reported on critical appraisal tools specific to one study design (such as the Cochrane risk-of-bias-tool for randomised trials) or review question (for instance, one paper reported on tools to review the acquired brain injury literature). Of the 78 records, 32 were screened on full text. We also screened the list of tools used by the National Institute for Health and Care Excellence (NICE) [3]. In addition, members of the COVID-19 Rapid Evidence Service (four Reviewers and one Information Scientist) identified any critical appraisal tools they considered relevant for this project.

Potentially relevant critical appraisal tools identified included checklists from the Joanna Briggs Institute (JBI) [4], the Critical Appraisal Skills Programme (CASP) [5] and the National Heart, Lung and Blood Institute (NHLBI) [6], as well as the Quality Criteria Checklist (QCC) [7] and the Mixed Methods Appraisal Tool (MMAT) [8]. The JBI [4], CASP [5], and NHLBI [6] tools are applicable to multiple study designs, including case series, but have one checklist for each study design. They therefore did not meet our criteria of having one single tool to assess a range of study designs.

The MMAT [8, 9] divides studies into five main categories (qualitative research, randomised controlled trials, non-randomised studies, quantitative descriptive studies, and mixed methods studies), with one checklist for each category. The QCC has one unified checklist applicable to all study designs [10]. Both of these tools were therefore potentially of use within our rapid reviews.

## Selection of a critical appraisal tool

The QCC is composed of 10 questions that requires responses of ‘yes, ‘no’, ‘unclear’ or ‘N/A’ (not applicable) [7, 10]. Each question includes sub-questions that identify important aspects to consider and specify how it should be applied to a specific study design. The MMAT consists of five questions which can be answered as ‘yes’, ‘no’ or ‘can’t tell’ [8].

Three reviewers, with different levels of experience, independently assessed four individual studies with both the MMAT and the QCC. The four studies reported on COVID-19 transmission but were of different design: one ecological study, one case report, one cross-sectional study and one retrospective cohort study. Agreement between the three reviewers was assessed using the Fleiss kappa coefficient, which showed good agreement for the QCC tool (*k* = 0.639) compared to only fair agreement for the MMAT tool (*k* = 0.261). The QCC contained a greater number of questions than the MMAT although the presence of sub-questions resulted in the reviewers finding the QCC clearer and more comprehensive than the MMAT. As a result, the reviewers felt more confident using the QCC tool than the MMAT tool and they found it easier to use. The time taken to complete was similar for both tools, usually ranging from 30 min to 1 h depending on the difficulty of the study, previous knowledge of the study (especially whether the reviewer had done the data extraction beforehand), and overall experience of the reviewer.

We agreed, based on these results, that the QCC tool would be used to critically appraise the studies included in all future COVID-19 rapid reviews. In a non-pandemic situation, it would have been preferable to compare agreement between tools using more studies, and to conduct qualitative research to record reviewers’ experience of using the tools in a more formal way. However, due to the speed of our work in this emergency pandemic context, this proved unfeasible. The clear difference in agreement and reviewers’ experience observed between tools enabled us to make the decision to use the QCC.

## Characteristics of the QCC

The 10 questions of the QCC are presented in the supplementary material (see Box 1, Additional file 1). The QCC tool was developed by the Academy of Nutrition and Dietetics and the questions are based on criteria and domains identified by the Agency for Healthcare Research and Quality (AHRQ) to assess the methodological quality of a study [11]. The QCC was developed for nutrition-related studies, but the questions are also relevant to the COVID-19 studies assessed for our rapid reviews as both rely mainly on epidemiological observational studies.

The rating of the methodological quality of the studies is based on a system of critical questions rather than a numerical score. In the QCC, the four questions considered as critical are question 2 on selection bias, question 3 on group comparability (not applicable in descriptive studies), question 6 on intervention/exposure assessment and question 7 on outcome assessment.

The rating terminology in the original QCC was ‘positive’, ‘neutral’ and ‘negative’, which we amended to ‘high’, ‘medium’ and ‘low’ (see box 2, Additional file 1). A study is rated as ‘high’ methodological quality if the answer to the four critical questions is ‘yes’, plus at least one of the non-critical questions. A study is rated as ‘low’ methodological quality if 50% or more of the critical questions answered ‘no’ (or more than 50% of the non-critical questions answered ‘no’). Otherwise, the study is rated as ‘medium’ methodological quality. Judgment on the rating is made on a case-by-case basis for questions answered as ‘unclear’.

As with any critical appraisal tool, the QCC tool should be used by reviewers with appropriate knowledge and training. In our rapid reviews, critical appraisal was usually conducted by one reviewer and checked by a second.

## Discussion and conclusion

We started using the QCC tool in January 2021, and it has been used in all our rapid systematic reviews completed since then, including six published on our website and one in the BMJ [2, 12]. Although the tool had originally been developed for assessment of evidence in the nutrition and dietetics field, the reviewers who have used it in our COVID-19 rapid reviews have found it relevant and reliable. Once the reviewers were familiar with the tool, it was easy and fast to apply, which is an important consideration when conducting a rapid review. As expected, being able to use one single tool across a range of experimental and observational studies was paramount to conducting those reviews at pace.

Our results suggest that the QCC is an appropriate tool for appraising experimental and observational studies included within COVID-19 rapid reviews, although further reliability analyses should be conducted. More research is also needed to validate the QCC across a range of different public health issues.

## Supplementary Information

**Additional file 1.** The list of questions and the quality ratings of the QCC are provided.

## Data Availability

The dataset supporting the conclusions of this letter is available from the corresponding author on reasonable request.

## Abbreviations

AHRQ: Agency for Healthcare Research and Quality
CASP: Critical Appraisal Skills Programme
JBI: Joanna Briggs Institute
MMAT: Mixed Methods Appraisal Tool
NHLBI: National Heart, Lung and Blood Institute
NICE: National Institute for Health and Care Excellence
N/A: Not applicable
PHE: Public Health England
QCC: Quality criteria checklist
UKHSA: UK Health Security Agency
